# Interaction of hnRNPA1/A2 and DAZAP1 with an *Alu*-Derived Intronic Splicing Enhancer Regulates *ATM* Aberrant Splicing

**DOI:** 10.1371/journal.pone.0023349

**Published:** 2011-08-08

**Authors:** Tibor Pastor, Franco Pagani

**Affiliations:** International Centre for Genetic Engineering and Biotechnology, Trieste, Italy; St. Georges University of London, United Kingdom

## Abstract

We have previously identified an *Alu*-derived Intronic Splicing enhancer (ISE) in the Ataxia Teleangectasia Mutated gene (*ATM*) that facilitates intron pre-mRNA processing and leads to the inclusion of a cryptic exon in the final mRNA transcript. By using an RNA pull-down assay, we show here that hnRNPA1/A2, HuR and DAZAP1 splicing factors and DHX36 RNA helicase bind to the ISE. By functional studies (overexpression and siRNA experiments), we demonstrate that hnRNPA1 and DAZAP1 are indeed involved in ISE-dependent ATM cryptic exon activation, with hnRNPA1 acting negatively and DAZAP1 positively on splicing selection. On the contrary, HuR and DHX36 have no effect on ATM splicing pattern. These data suggest that splicing factors with both negative and positive effect can assemble on the intronic *Alu* repeats and regulate pre-mRNA splicing.

## Introduction

Accurate intron excision and exon joining during the process of pre-mRNA splicing, enable generation of mature mRNAs that contain continuous coding sequence for protein synthesis. Precise pre-mRNA splicing depends on the presence of splicing consensus sequences at 5′ and 3′ exon splice sites and additional intronic and exonic regulatory elements [1]. These elements are defined as Splicing Enhancer or Silencers according to their effect on splice site selection and are involved in normal and aberrant splicing regulation [2]. Differently from classical splice sites, enhancers and silencers are highly degenerated sequences that affect splicing through their interaction with regulatory splicing factors and/or RNA secondary structure [1]. RNA-binding factors are key splicing regulators as their interaction with intronic and/or exonic sequences contributes to the splicing outcome. In general, each splicing factor has a positive or a negative effect on splicing, for example SR proteins are considered enhancers whereas hnRNPA1/A2 are silencers, but frequently the final effect on splicing is based on their binding position [3], which reflects specific interactions with multiple spliceosomal components on the nascent transcripts.

Genomic variants located in “deep” intronic regions, far away from exon/intron junctions, may alter the pattern of splicing leading to generation of aberrantly spliced products. In the majority of cases, these mutations affect the classical *cis*-acting elements required for exon definition like the splice sites sequences [4]. However, in some cases, mutations may affect different splicing regulatory elements that are important for intron processing. We previously reported a particular four nucleotides-long GTAA deletion in the ATM intron 20, which affects normal intron processing: it activates a cryptic exon inclusion in the final mRNA transcript and cause ataxia telangiectasia in affected patients [5]. Activation of the cryptic exon is a result of a complex mechanism that includes several *cis*-acting elements, which are involved in aberrant processing of the intron. In fact, this mutation occurs 12 bp downstream and 53 bp upstream of the cryptic exon splice sites, respectively, and activates a weak cryptic GC 5′ splice site. Moreover, this mutation leads to the disruption of an Intronic Splicing Processing Element (ISPE) that is located between these two cryptic sites: the GTAA deletion actually abolishes the non-canonical interaction of U1 snRNP with the ISPE [5]. This further leads to a stringent 5′-3′ order of intron removal around the ATM cryptic exon by promoting the splicing of the upstream intron due to the removal of the steric hindrance from the adjacent upstream cryptic 3′ splice site [6], [7]. This eventually results in generation of the 5′ splicing intermediate that contains the downstream portion of the ATM intron 20. Interestingly, the splicing defect is modulated by an *Alu*-derived Intronic Splicing Enhancer (ISE), that is indispensable for the inclusion of the cryptic exon in the final mRNA transcript [8]. This 40-nt long ISE element resides within an inverted *Alu* repeat situated downstream of the cryptic exon thus suggesting an interesting impact of this primate-specific mobile element in modulation of ATM cryptic exon activation. Actually, the effect of *Alu* repeats on the pre-mRNA splicing is relevant for human pathology and primate-specific evolution as their intronic insertion has already been associated with pathological skipping of adjacent exons in several human diseases [9], [10]. Furthermore, genome-wide analyses showed that many *Alu* repeats preferentially flank alternatively spliced exons rather than constitutively spliced ones thus implying that *Alu* repeats may change the mode of splicing of the flanking exons and affect the splicing outcome [11]. The ISE in the ATM intron 20 acts in a peculiar manner facilitating intron pre-mRNA processing of the aberrant transcript. In fact, whereas the excision of the upstream portion of ATM intron 20 and generation of the 5′ splicing intermediate occur regardless of the presence of the ISE, further processing of the precursor is ISE-dependent [8]. Intermediates that lack this regulatory element do not give rise to the mature mRNA that contains the cryptic exon. The ISE-mediated processing of the 5′ splicing intermediate is also related to the inefficient recognition of the weak cryptic GC 5′ splice site by U1 snRNP [8].

To better understand the function of the *Alu*-derived ISE in intron processing, we explored its binding properties towards *trans*-acting factors. We found that several splicing factors specifically bind to the ISE. Functional analysis with over-expression and siRNA experiments showed that the ISE-mediated cryptic exon activation involves hnRNPA1 and DAZAP1 *trans*-acting factors whose interaction with this regulatory element modulates ATM splicing outcome. As the human genome harbors a large amount of similar *Alu*-derived regulatory elements, it suggests that their potential interaction with these *trans*-acting factors may represent a widespread phenomenon with effect on pre-mRNA processing and alternative splicing regulation.

## Results

### Identification of nuclear proteins that differentially bind to the ISE wt and ISE mutated sequences

**Figure 1:**
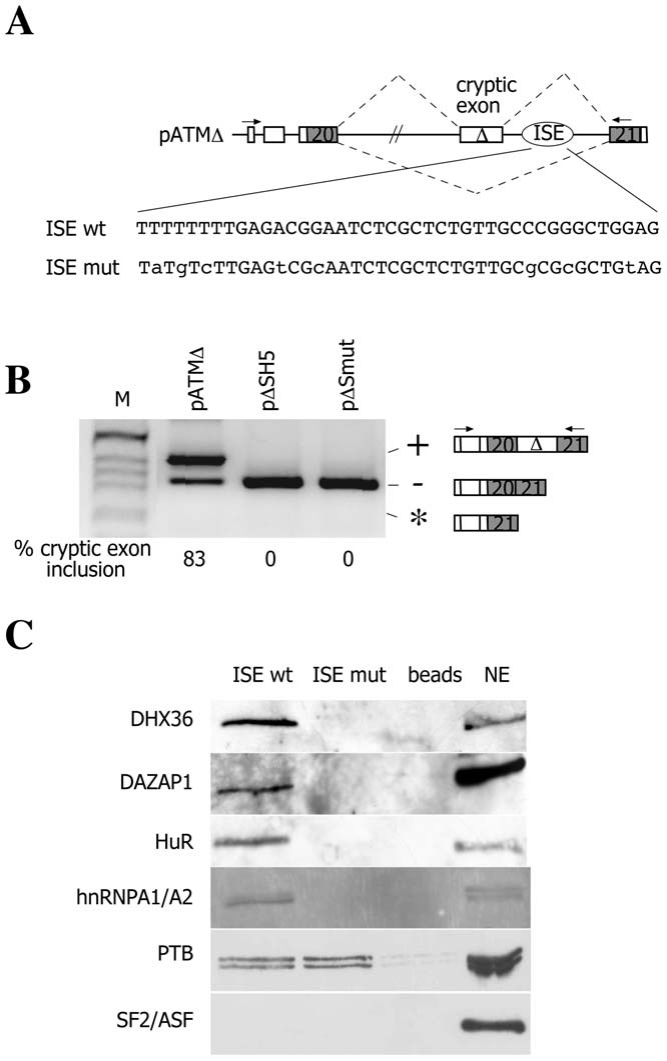
Analysis of the ISE binding capacity. A) Schematic representation of the pATM minigene. α-globin and ATM exons are white and grey boxes, respectively, and introns are lines. The cryptic 65 bp ATM exon activated by the GTAA deletion (Δ) is indicated and the oval show the position of the ISE. Splicing pattern is represented with diagonal dashed lines and the arrows indicate location of the primers used in RT-PCR analysis. The nucleotide sequence of normal and mutant ISE is shown. B) Splicing assay. The pΔ minigenes were transfected in HeLa cells and the pattern of splicing analyzed with E16 and 2550 primers. In pΔSH5 the ISE has been deleted. RT-PCR fragments were resolved on 2% agarose gel. M is the molecular weight marker. The cryptic exon inclusion and exclusion are indicated. The asterisk corresponds to a minor spliced product without the hybrid exon made of globin exon 3 and ATM exon 20. The % of cryptic exon inclusion is indicated and is the mean of three independent experiments. C) Western blot analysis of pull down assay performed on ISE wt and ISE mut sequences. The binding analysis of RNA helicase DHX36, DAZAP1, HuR, hnRNPA1, PTB and SF2/ASF protein. Upon *in vitro* transcription, RNAs were covalently bound to the agarose beads and incubated with HeLa nuclear extract. After washing steps, the pulled down proteins were loaded on SDS-PAGE gel and analyzed by western blotting using antibodies against RNA helicase DHX36, DAZAP1, HuR, hnRNPA1, PTB and SF2/ASF proteins. The nuclear extract (NE) corresponds to the 1/20 of the amount used in pull down assay. ISE wt sequence pulled down RNA helicase DHX36, DAZAP1, HuR, hnRNPA and PTB whereas the ISE mut fraction was enriched only in PTB. SF2/ASF was not detected in either of fractions.

The basic pATMΔ hybrid minigene construct comprised the entire sequence of ATM intron 20 flanked by ATM exons 20 and 21 and embedded in the α-globin context (Fig. 1A). As previously reported, pATMΔ construct, which contains the four bp deletion found in patient, generates an aberrantly spliced product with ∼83% of cryptic exon inclusion whereas removal of the downstream ISE restores normal pattern of splicing (Fig. 1B, pΔSH5). Similarly, the introduction of eight nucleotide changes across the ISE also leads to the complete exclusion of the cryptic exon from the mature mRNA transcript (Fig. 1B, pΔmut).

To identify specific *trans*-acting factors whose differential binding to the wild type and mutated regulatory element could provide the explanation for observed changes in the splicing pattern, we carried out a pull down assay using the ISE wt and ISE mutated element. The pull down experiment revealed several protein bands exclusively enriched in ISE wt and not in the ISE mut oligonucleotide fraction. These protein bands were excised from gel, sequenced performing electrospray mass spectrometry analysis and eventually identified as ATP-dependent RNA helicase DHX36 (Uniprot accession number Q9H2U1), DAZAP1 (Deleted in AZoospermia-Associated Protein 1, Uniprot accession number Q96EP5) and HuR (Hu-antigene R, Uniprot accession number Q15717) proteins, respectively. To confirm the differential binding capacity, immunoblot experiment was performed including in the analysis some proteins whose role in splicing regulation is well described in the literature: hnRNPA1 and PTB proteins as well as SF2/ASF protein. The immunoblot analysis confirmed the presence of RNA helicase DHX36, DAZAP1 and HuR proteins only in the ISE wt fraction and revealed that also hnRNPA1 protein was enriched in ISE wt fraction (Fig. 1C). PTB protein was present in equal amounts in both ISE wt and ISE mut samples while SF2/ASF protein had no binding affinity towards either of the two sequences. Altogether these results indicate that RNA helicase DHX36, DAZAP1, HuR and hnRNPA1 proteins specifically interact *in vitro* with the ISE.

### Effect of RNA helicase DHX36, DAZAP1, HuR and hnRNPA1 overexpression on ATM cryptic exon activation

To address the functional role of these *trans*-acting factors in ATM cryptic exon activation, we performed cotransfecton experiments using minigene constructs with and without the ISE (pATMΔ and pΔSH5, respectively). These constructs were transfected into HeLa cells alone or along with expression vectors for DHX36, DAZAP1, HuR and hnRNPA1 proteins whose effect on the minigene splicing pattern was evaluated by RT-PCR. Expression of all four recombinant proteins was confirmed by conducting the western blot analyses using anti-flag antibody (Fig. S1).

**Figure 2:**
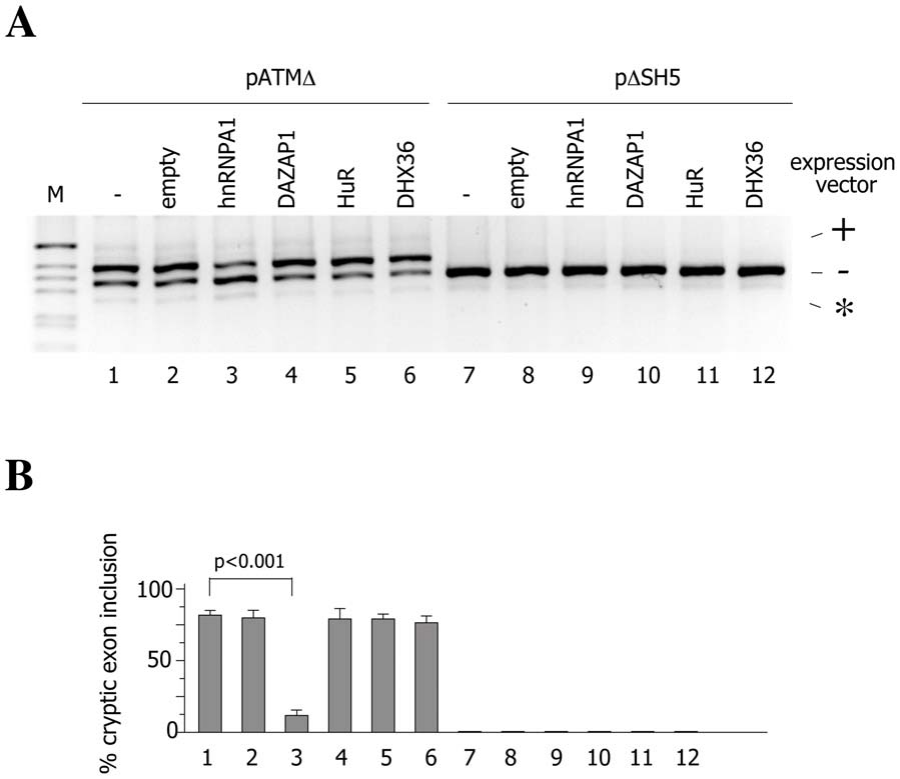
Effect of RNA helicase DHX36, DAZAP1, HuR and hnRNPA1 overexpression on ATM cryptic exon activation of pATMΔ and pΔSH5 minigenes. (A) HeLa cells were transfected with pATMΔ and pΔSH5 minigenes in presence of empty expression vector (lanes 2 and 8, respectively) or vectors expressing the protein of interest (lanes 3-6 and 9-12, respectively). The splicing pattern was analyzed with the E16 and 2550 primers. RT-PCR fragments were resolved on 2% agarose gel. M is the molecular weight marker. On the right is indicated the splicing product with (+) or without (-) cryptic exon inclusion. The asterisk corresponds to a minor spliced product without the hybrid exon made of globin exon 3 and ATM exon 20. (B) The graph is the quantification of three independent experiments expressed as means ±SD.

**Figure 3:**
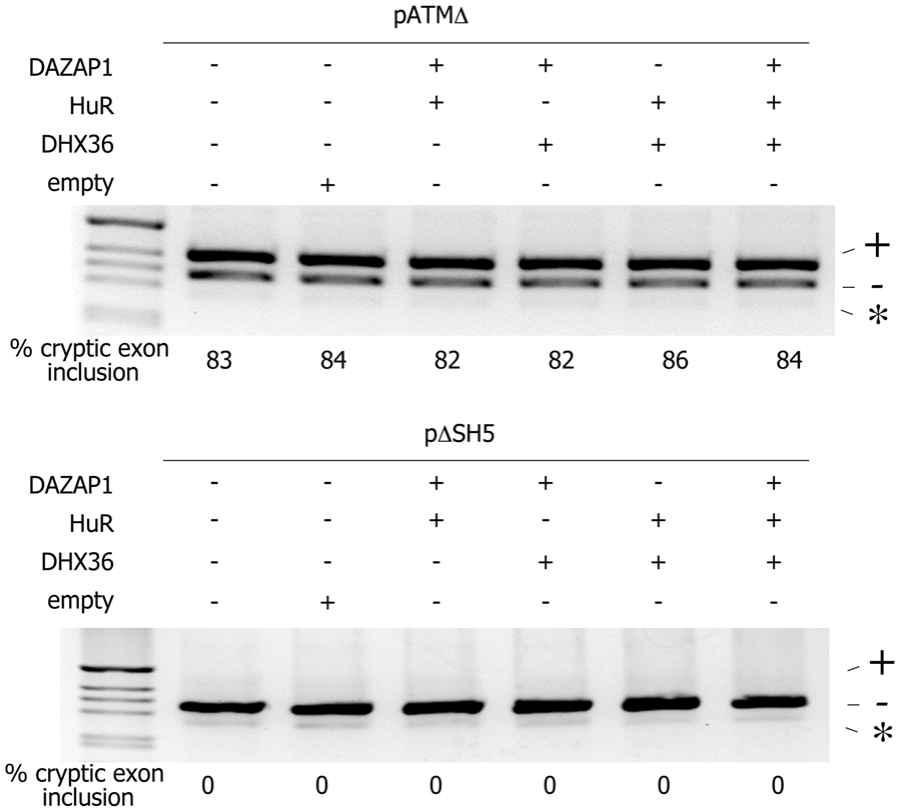
Effect of RNA helicase DHX36, DAZAP1 and HuR co-expression on ATM cryptic exon activation of pATMΔ and pΔSH5 minigenes. (A) HeLa cells were transfected with pATMΔ minigene in presence of empty expression vector (lanes 2) or different combinations of vectors expressing the RNA helicase DHX36, DAZAP1 and HuR protein of interest (lanes 3-6). The splicing pattern was analyzed with the E16 and 2550 primers. RT-PCR fragments were resolved on 2% agarose gel. M is the molecular weight marker. On the right is indicated a splicing product with (+) or without (-) cryptic exon inclusion. The asterisk corresponds to a minor spliced product without the hybrid exon made of globin exon 3 and ATM exon 20. The % of cryptic exon inclusion is indicated and is the mean of two independent experiments. (B) HeLa cells were transfected with pΔSH5 minigene in presence of empty expression vector (lane 2) or different combinations of expression vectors for RNA helicase DHX36, DAZAP1 and HuR protein (lanes 3-6). The splicing pattern was analyzed with E16 and 2550 primers. RT-PCR fragments were resolved on 2% agarose gel. M is the molecular weight marker. On the right is indicated a splicing product with (+) or without (-) cryptic exon inclusion.

The splicing of the ISE-containing pre-mRNA construct (pATMΔ) was affected only by the overexpression of hnRNPA1 protein whereas the overexpression of other candidate proteins had no effect on the pattern of splicing (Fig. 2). In particular, hnRNPA1 overexpression led to a decrease of cryptic exon inclusion from 80% to 10% in pATMΔ (Fig. 2, lane3). On the other hand, pΔSH5 construct that lacks the ISE element did not respond to the increased amount of any of proteins tested (Fig. 3, lanes 7–12). In order to see whether RNA helicase DHX36, DAZAP1 and HuR protein can induce some effect on ATM cryptic exon inclusion upon co-expression, we performed the experiment in which overexpression of these three proteins was combined in different manners. Neither overexpression of two different proteins in all three combinations nor simultaneous overexpression of HuR, DAZAP1 and DHX36 showed any effect on tested minigene constructs (Fig. 3). In addition, the decrease of cryptic exon inclusion induced by hnRNPAI in pATMΔ was not affected by the simultaneous coexpression of the other splicing factors (data not shown).

### Effect of siRNA against RNA helicase DHX36, DAZAP1, HuR and hnRNPA1 on ATM cryptic exon activation

In order to evaluate the functional role of RNA helicase DHX36, DAZAP1, HuR and hnRNPA1/A2 on ATM cryptic exon activation, we depleted these proteins from cells by performing siRNA treatment.

**Figure 4:**
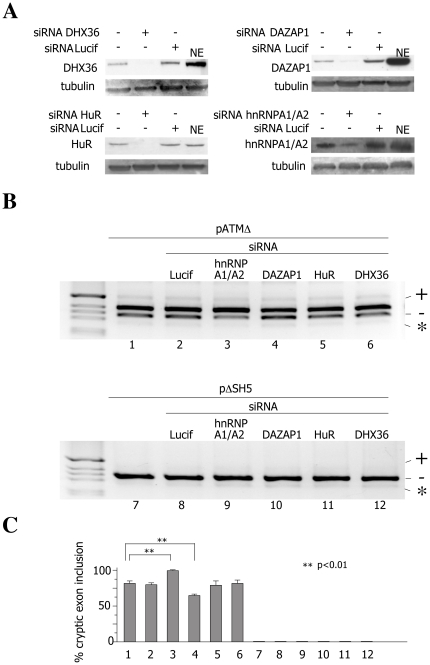
Depletion of hnRNPA1 and DAZAP1 affects splicing of ISE‐containing pATMΔ construct. A) Western blot analysis of HeLa cells treated with siRNA oligonucleotides against RNA helicase DHX36, DAZAP1, HuR and hnRNPA1 protein. siRNA against luciferase was used as a control. The western blot was carried out using antibodies that were specific for each target protein. Tubulin was used as a control of the amount of protein loaded on gel. NE stands for nuclear extract. The siRNA experiment is representative of two independently performed experiments. Protein depletion observed was almost complete. B) siRNA-treated and untreated HeLa cells were transfected with pATMΔ and pΔSH5 minigene constructs and the splicing pattern was analyzed by RT-PCR. RT-PCR fragments were resolved on 2% agarose gel. M is the molecular weight marker. The resulting splicing products with cryptic exon inclusion (+) or exclusion (-) are indicated. The identity of siRNAs that were used to knock‐down corresponding proteins is indicated. C) The graph is the quantification of three independent experiments expressed as means ±SD

*In vivo* depletion of DHX36, DAZAP1, HuR and hnRNPA1/A2 proteins was achieved by transient transfection of siRNA oligonucleotides directed toward corresponding proteins. All siRNA treatments induced significant reduction of the target protein level (Fig. 4A). Silencing of DHX36, HuR and DAZAP1 was complete while siRNA against hnRNPA1/A2 led to 90% of protein reduction. The cells treated with different siRNAs were transfected with pATMΔ and pΔSH5 minigene constructs. The siRNA treatment against hnRNPA1 led to an increase in the ATM cryptic exon inclusion up to 95% in pATMΔ context (Fig. 4B, lane 3) but had no effect on pΔSH5 constructs (Fig. 4B, lane 9). Depletion of DAZAP1 induced a mild ATM cryptic exon exclusion in pATMΔ-deriving mature mRNA transcripts (Fig. 4B, lane 4) and had no effect on transcripts that lack the ISE (Fig. 4B, lane10). The siRNA treatments against RNA helicase DHX36 and HuR had no effect on either of tested pATM minigenes regardless of the presence of the ISE (Fig. 4B). Altogether these data demonstrate a functional role for hnRNPA1/A2 and DAZAP1 protein in ATM cryptic exon activation and show that these *trans*-acting factors have an opposite effect on ISE-mediated cryptic exon inclusion, with hnRNPA1/A2 promoting the cryptic exon exclusion and DAZAP1 facilitating cryptic exon inclusion in the final mRNA.

## Discussion

Genomic variants that affect splicing regulatory elements can alter the normal pattern of splicing and consequently cause or modify the severity of human diseases. Their effect depends on the complex interplay between *cis*-acting elements, which reside both in exonic and intronic regions, and *trans*-acting factors that they interact with. However, the function of intronic sequences in modulation of pre-mRNA splicing is poorly understood so the analyses of intronic mutations can help in elucidating the intronic determinants of the splicing code. The deletion of 4 bp (GTAA) within the ATM intron 20 has previously been reported to affect the process of splicing, leading to the ATM cryptic exon activation and eventually generating an aberrantly spliced mature mRNA. This deep intronic GTAA deletion in the ATM gene differs from the majority of described intronic variants as it is not directly related to the changes at splice sites but instead affects an Intronic Splicing Processing Element (ISPE) whose disruption then abolishes a non-canonical interaction with U1 snRNP and consequently leads to the activation of two nearby cryptic splice sites [5]. However, this mutation *per se* does not give rise to the aberrant mature RNA transcript as its production depends on the downstream *Alu*-derived ISE. To test the hypothesis that ISE-dependent cryptic exon activation requires interaction of the ISE with *trans*-acting factors, the binding capacity of the ISE sequence was evaluated and four proteins with binding affinity towards ISE element were detected: RNA helicase DHX36, DAZAP1, hnRNPA1 and HuR (Fig. 4). HuR is an RNA-binding protein that shuttles between the cytoplasm and the nucleus and binds with high affinity to AU-rich elements (AREs) [12]. HuR was originally reported to be implicated in stabilization of ARE-containing mRNAs but it has been recently shown that is also involved in splicing regulation where it promotes Fas exon 6 skipping by binding to an exonic splicing silencer [13]. Recent work also reports an extensive association of HuR with hnRNP proteins within the mRNP complex in the nucleus and cytoplasm suggesting a very important role of HuR in mRNA processing [14]. A few data are available for RNA helicase DHX36 that has been found to be involved in degradation and deadenylation of mRNAs containing ARE sequence element in their 3′-UTR [15]. This substrate “specificity” is mediated indirectly by its RNA-dependent interaction with the ARE-binding proteins HuR and NFAR1 and it is believed that RNA helicase DHX36 might actually serve as a “molecular motor” to drive the mechanics of complex RNA remodeling/decay reactions through interactions with HuR and NFAR proteins [15]. Interestingly, there exist two different isoforms of RNA helicase DHX36, which are generated through alternative splicing and that differ in an 8-amino acids long sequence that encodes for a nuclear localization signal (NLS). The RNA helicase DHX36 isoform that we detected in pull down experiments contains the NLS thus suggesting possible role of this protein in nuclear mRNA processing. DAZAP1 is involved in mRNA transport, stability and translational regulation [16]. This protein was shown to interact with many members of hnRNP family of proteins including the hnRNPA1 protein [17]. Considering that these two proteins are shuttling proteins, their interaction is considered to be important not only for mRNA export but also for splicing. In fact, DAZAP1 together with hnRNPA1/A2 binds to an exonic splicing silencer and promotes skipping of BRCA1 exon 18 thus revealing the role of DAZAP1 in pre-mRNA splicing regulation [18]. hnRNPA1 is a well-characterized splicing factor that exhibits an inhibitory role on the pre-mRNA splicing process [19]. As mentioned above, hnRNPA1 interaction with the other two shuttling proteins and splicing factors, HuR and DAZAP1, has been demonstrated to occur extensively both in the cytoplasm and the nucleus thus indicating their functional link in mRNA metabolism.

To address the functional role of these four proteins in ATM cryptic exon activation, we performed overexpression experiments and siRNA treatments on ISE-containing construct (pATMΔ) and constructs without the ISE (pΔSH5). While the overexpression of hnRNPA1 led to a diminished level of cryptic exon inclusion in ISE-containing constructs (Fig. 2), no effect was observed on either of tested constructs upon overexpression of RNA helicase DXH36, DAZAP1 and HuR (Fig. 2). Additionally, no changes in splicing pattern of tested minigenes were detected upon coexpression of RNA helicase DXH36, DAZAP1 and HuR (Fig. 3). On the other hand, depletion of candidate proteins revealed that hnRNPA1 and DAZAP1 induced changes in ATM cryptic exon inclusion on ISE-containing constructs (Fig. 4) whereas RNA helicase DHX36 and HuR depletion did not affect the cryptic exon inclusion in neither of tested constructs. In fact, the siRNA treatment against hnRNPA1/A2 and DAZAP1 had an opposite effect on cryptic exon activation as depletion of hnRNPA1 led to an increase in cryptic exon inclusion while DAZAP1 knock down induced a modest increase in cryptic exon exclusion suggesting that hnRNPA1/A2 and DAZAP1 proteins regulate ATM cryptic exon inclusion probably in an ISE-dependent manner.

The observation that hnRNPA1/A2 proteins acts to inhibit ATM cryptic exon inclusion is consistent with its negative role in pre-mRNA processing, while the DAZAP1 enhancing effect on ATM cryptic exon inclusion represents a new finding as this protein was previously described to perform exclusively as a splicing inhibitor [18], [20]. The antagonistic effect of hnRNPA1/A2 and DAZAP1 splicing factors points out a complex interplay between positive and negative factors in ATM cryptic exon inclusion. Although a direct proof of competitive interaction of these proteins with the ISE is missing, it is possible that hnRNPA1/A2 and DAZAP1 proteins compete for the same target sequence. However, an alternative explanation for the ISE-mediated antagonistic effect of hnRNPA1/A2 and DAZAP1 on cryptic exon activation could be that the ISE represents a more complex regulatory element that contains both enhancing and silencing sequences, whose function can be modulated by ISE-flanking sequences or affected by ISE-secondary structure determinants. Nevertheless, considering that hnRNPA1/A2 and DAZAP1 are abundant cellular proteins, that ISPE deletion in ISE context leads to 85% of cryptic exon inclusion and that depletion of DAZAP1 has only a modest effect on cryptic exon exclusion, it is more likely that other still unknown *trans*-acting factors are implicated in ISE-dependent enhancement of ATM cryptic exon inclusion in the mature mRNA. The apparent discrepancy between the overexpression and the knockdown experiments of DAZAP1 may be simply due to the fact that this splicing factor is an abundant cellular protein present at saturating concentration *in vivo*. Thus, a further increase in DAZAP1 level will not affect splicing. We observed previously a similar case where the splicing pattern was largely unresponsive to overexpression of a splicing factor but very sensitive to its depletion [21]. However, since recent reports demonstrate that some short non-coding nuclear RNAs can be implicated in modulation of the pattern of splicing through interaction with their target sequences within the pre-mRNAs [22], we cannot exclude the possibility that these small regulatory non-coding RNA transcripts can also interact with the *Alu*-deriving ISE element and contribute to the ATM cryptic exon activation. Despite the fact that functional assays did not show any effect of RNA helicase DHX36 and HuR on cryptic exon inclusion, involvement of these proteins in regulation of cryptic exon activation cannot be completely ruled out as it might be that their effect on processing of ATM intron 20 and aberrant cryptic exon activation is compensated by other proteins that are yet to be identified.

In conclusion, we have identified two splicing factors, hnRNPA1/A2 and DAZAP1, that regulate ATM cryptic exon inclusion in an ISE-dependent manner. These splicing factors might be part of a large proteins complex that is assembled on intronic *Alu*-derived splicing regulatory elements to regulate pre-mRNA processing.

## Materials and Methods

### Analysis of the hybrid minigene expression

pATMΔ, pΔSH5 and pATMΔmut minigene constructs have been previously described [6], [8]. HeLa cells (2×10^6^) (ATCC, Cat. No. CCL-2) were grown in standard conditions and transfected with 500 ng of each minigene plasmid using Effectene reagent [18]. RNA extraction and RT-PCR of amplified products were done as previously described [23]. Quantification of the percentage of exon inclusion was performed on Et-Br agarose gels images using ImageJ 1.38 software (http://rsb.info.nih.gov/ij/) [23]. For the analysis of spliced forms pATM minigenes were amplified with E16 dir and ATM 2550 rev [8]. For cotransfection experiments HeLa cells were transfected with 500 ng of the minigene construct together with 500 ng of empty pCMVFlag expression vector or pCMV vector expressing the Flag-tagged proteins (HuR, DAZAP1, hnRNPA1 and DHX36 helicase). The expression of all four recombinant proteins was confirmed by western blot analysis using anti-flag antibody.

### Affinity purification of RNA-binding proteins (pull-down analysis) and mass spectrometry

The synthetic oligonucleotides were purchased from Sigma, annealed and cloned in pBluescript KS vector under the T7 RNA promoter. The sequences of oligos are listed in the Table S1. *In vitro* transcription was carried out with T7 RNA Polymerase (Promega) according to standard procedure. Approximately 10 µg of *in vitro* transcribed and purified RNA was placed in 400 µl of reaction mixture (100 mM NaOAc pH 5.0 and 5 mM sodium m-periodate), incubated in the dark at room temperature for 1 hour, precipitated with ethanol and finally resuspended in 100 µl of 100 mM NaOAc pH 5.0. In the meantime, 400 µl of adipic acid dehydrazide agarose beads 50% slurry were equilibrated with 100 mM NaOAc (pH 5.2), The beads were then added to each periodate-treated RNA and the mixture was incubated on a rotator at 4°C for 12 hours. The complex RNA-beads was then washed three times with 1 ml of 2 M NaCl, equilibrated in 1x Solution D (20 mM HEPES pH 7.5, 100 mM KCl, 0.2 mM EDTA, 0.5 mM DTT and 6% glycerol) and incubated, in the final volume of 500 µl, with 0.5 mg of HeLa cell nuclear extract (C4, Biotech), 10x solution D without KCl (200 mM HEPES pH 7.5, 2 mM EDTA, 5 mM DTT, and 60% glycerol), 160 mM KCl, and Heparin (final concentration 2.5 µg/µl), on a rotator at room temperature for 30 minutes. The beads were then washed six times with 1 ml of 1x solution D before the addition of SDS sample buffer and loading on 12% SDS-polyacrylamide gels. Gels were stained with Coomassie brilliant blue R250. The protein bands, which were differentially enriched in the ISE wt and the ISE mutated fractions, were excised from the gel and subjected to the protein sequence analyses by applying the electrospray ionization mass spectrometer (LCQ DECA XP-ThermoFinnigam). Fragments were then analyzed by mass spectrometry and proteins were identified by analysis of the peptide MS/MS data with Turbo SEQUEST (Thermo Finnigam) and MASCOT (Matrix Science). The primary antibodies that were applied in western blotting analysis are: rabbit polyclonal anti-hnRNPA1 antibody (Santa Cruz), rabbit polyclonal anti-DAZAP1 antibody (kindly provided by E. Goina, ICGEB, Italy; 1∶1000), goat polyclonal anti-HuR (Santa Cruz, 1∶800) and rabbit polyclonal anti-DHX36 (Bethyl Laboratories, 1∶1000).

### Small interfering (siRNA) transfection

siRNA transfections were performed in HeLa cells using Oligofectamine Reagent (Invitrogen). The sense strands of RNAi oligos (Dharmacon and Sigma), which were used to silence the target genes, are listed in the Table S2. 2.5×10^5^ HeLa cells were plated in 60 mm plates and two rounds of transient transfection with siRNA oligonucleotides against the proteins of interest were performed. 24 hours after the second treatment with siRNA the cells were transfected with the pATMΔ and pΔSH5 minigene constructs using Qiagen Effectene transfection reagents. After an additional 24 hours the cells were collected and divided in two equal fractions. The first one was used to extract the total RNA and evaluate the effect of protein depletion on the splicing pattern whereas the second fraction served to perform protein analysis in order to evaluate the silencing effect of siRNAs.

## Supporting Information

Figure S1 **Over-expression of HuR, DAZAP1, hnRNPA1 and RNA helicase DHX36 proteins in HeLa cells.** Western blot analysis on HeLa cells non-transfected (−) and transfected (+) with expression vectors for HuR, DAZAP1, hnRNPA1 and RNA helicase DHX36. The proteins were tagged with flag and their expression was detected using corresponding antibody.
(TIF)

Table S1 Oligonucleotide sequence for mutagenesis experiments.
(DOC)

Table S2 Sequence of RNAi oligonucleotides.
(DOC)
