# Supplementary figures and images for: Interaction of hnRNPA1/A2 and DAZAP1 with an Alu-Derived Intronic Splicing Enhancer Regulates ATM Aberrant Splicing
Source: PLoS One. 2011 Aug 8;6(8):e23349. doi: 10.1371/journal.pone.0023349 (PMC3152568; doi:10.1371/journal.pone.0023349)

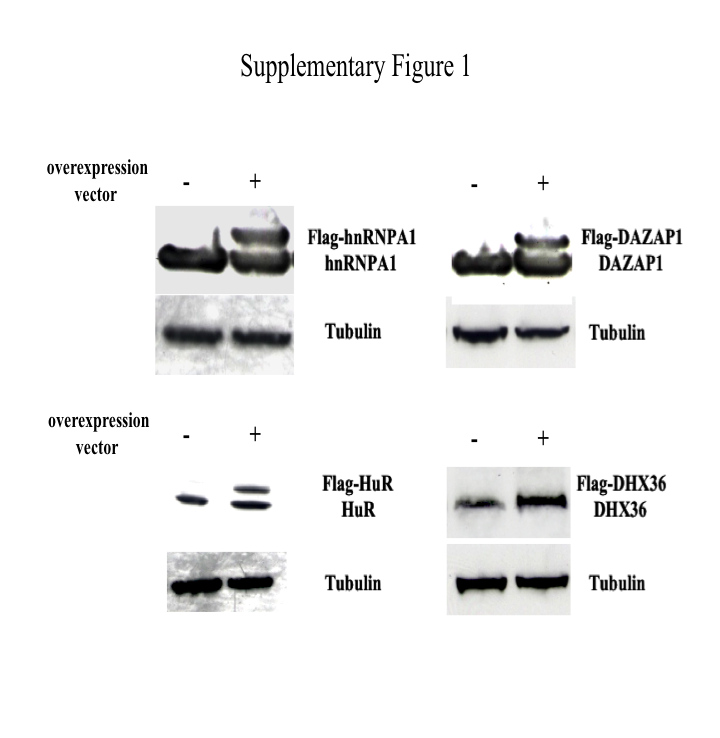

Supplement: Figure S1 — Over-expression of HuR, DAZAP1, hnRNPA1 and RNA helicase DHX36 proteins in HeLa cells. Western blot analysis on HeLa cells non-transfected (−) and transfected (+) with expression vectors for HuR, DAZAP1, hnRNPA1 and RNA helicase DHX36. The proteins were tagged with flag and their expression was detected using corresponding antibody. (TIF) [file pone.0023349.s001.tif]
