# Supplementary material for: Interaction of hnRNPA1/A2 and DAZAP1 with an Alu-Derived Intronic Splicing Enhancer Regulates ATM Aberrant Splicing
Source: PLoS One. 2011 Aug 8;6(8):e23349. doi: 10.1371/journal.pone.0023349 (PMC3152568; doi:10.1371/journal.pone.0023349)
Supplement: Table S1 — Oligonucleotide sequence for mutagenesis experiments. (DOC) [file pone.0023349.s002.doc]

**Table S1**

| Name of the oligo | Sequence of the oligonucleotide |
| --- | --- |
| Sh31 for | 5’-GATCTTTTTTTTTGAGACGGAATCTCGCTCTGTTGCCCGGGCTGGAGT-3’ |
| Sh31 rev | 5’-GATCACTCCAGCCCGGGCAACAGAGCGAGATTCCGTCTCAAAAAAAAA-3’ |
| Sh31 mut for | 5’-GATCTTATGTCTTGAGTCGCAATCTCGCTCTGTTGCGCGCGCTGTAGT-3’ |
| Sh31 mut rev | 5’-GATCACTACAGCGCGCGCAACAGAGCGAGATTGCGACTCAAGACATAA-3’ |
