# Supplementary material for: Interaction of hnRNPA1/A2 and DAZAP1 with an Alu-Derived Intronic Splicing Enhancer Regulates ATM Aberrant Splicing
Source: PLoS One. 2011 Aug 8;6(8):e23349. doi: 10.1371/journal.pone.0023349 (PMC3152568; doi:10.1371/journal.pone.0023349)
Supplement: Table S2 — Sequence of RNAi oligonucleotides. (DOC) [file pone.0023349.s003.doc]

**Table S2**

| Name of the oligo | Sequence of the sense strand of RNAi oligonucleotide |
| --- | --- |
| hnRNPA1 | 5’-CAGCUGAGGAAGCUCUUCA-3’ |
| hnRNPA2 | 5’-GGAACAGUUCCGUAAGCUC-3’ |
| DAZAP1 | 5’-GAGACUCUGCGCAGCUACU-3’ |
| HuR | 5’-AAGAGGCAAUUACCAGUUUCA-3’ |
| DHX36 helicase | 5’-GGGAACUGCGAAGAAGGUA-3’ |
| Luciferase | 5’-GCCAUUCUAUCCUCUAGAGGAUG-3’ |
